# Prenatal Diagnosis of Congenital Heart Disease: The Crucial Role of Perinatal and Delivery Planning

**DOI:** 10.3390/jcdd11040108

**Published:** 2024-03-31

**Authors:** Sheetal R. Patel, Erik Michelfelder

**Affiliations:** 1 Ann & Robert H Lurie Children’s Hospital of Chicago, Feinberg School of Medicine, Northwestern University, Chicago, IL 60611, USA; 2 Children’s Healthcare of Atlanta, Emory School of Medicine, Emory University, Atlanta, GA 30265, USA

**Keywords:** fetal echocardiography, congenital heart defects, delivery planning, perinatal management

## Abstract

Although most congenital heart defects (CHDs) are asymptomatic at birth, certain CHD lesions are at significant risk of severe hemodynamic instability and death if emergent cardiac interventions are not performed in a timely fashion. Therefore, accurate identification of at-risk fetuses and appropriate delivery resource planning according to the degree of anticipated hemodynamic instability is crucial. Fetal echocardiography has increased prenatal CHD detection in recent years due to advancements in ultrasound techniques and improved obstetrical cardiac screening protocols, enabling the prediction of newborns’ hemodynamic status. This assessment can guide multidisciplinary resource planning for postnatal care, including selection of delivery site, delivery room management, and transport to a cardiac center based on CHD risk severity. This review will discuss fetal cardiovascular physiology and the circulatory changes that occur at the time of and immediately following birth, outline fetal echocardiographic findings used to risk-stratify newborns with CHDs, and outline principles for neonatal resuscitation and initial transitional care in neonates with these complex CHD lesions.

## 1. Background

Congenital anomalies of the heart (i.e., CHDs) are the most common congenital organ system anomaly, occurring in approximately 1 out of 110 live births [1,2]. With advances in diagnostic imaging and improved obstetrical ultrasound guidelines [3], prenatal diagnosis of CHDs has improved in recent years [4,5,6]. Accurate prenatal assessment by fetal echocardiography and individualized, echocardiography-based perinatal management plans have improved both pre- and postnatal clinical outcomes in the CHD population [7,8,9]. A multidisciplinary team strategy that includes collaboration amongst fetal cardiologists, maternal–fetal medicine specialists, neonatologists, and other clinical services is ideal for implementing effective perinatal management plans for newborns with prenatal diagnosis of CHDs [8]. Based on the fetal echocardiographic findings, risk-stratification guidelines for CHDs have been developed based on the specific CHD and the expected degree of postnatal hemodynamic compromise [8,10,11] (Table 1). This review aims to summarize the delivery planning guidelines for perinatal management of CHDs based on the predicted severity of postnatal hemodynamic instability. These guidelines are useful in establishing recommendations for appropriate delivery sites, modes of delivery, immediate postnatal care, and anticipating the need for urgent cardiac interventions soon after birth.

### 1.1. Fetal Circulation in the Presence of CHDs and Transition at the Time of Birth

**Figure 1:**
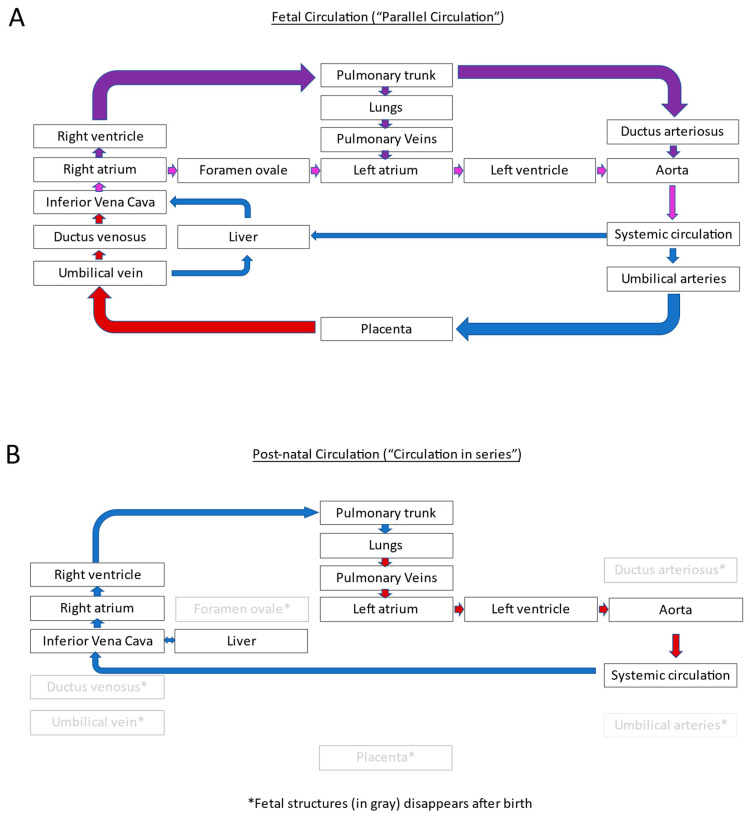
Schematic diagram showing normal fetal circulation (**A**) and postnatal circulation (**B**). Closure of the fetal shunts (ductus arteriosus, ductus venosus, and foramen ovale) and separation from the placenta cause the transition of fetal circulation to postnatal circulation. Blue arrows indicate deoxygenated blood, red arrow indicate oxygenated blood, purple arrows indicated mixed blood.

In the fetus, circulation is characterized by a “parallel circulation”, where the right and left ventricles handle their fraction of the combined fetal cardiac output in the presence of fetal shunts, including the ductus venosus, foramen ovale, and ductus arteriosus (Figure 1A). At birth, removing the low-resistance placental circulation at cord clamping results in an acute increase in systemic vascular resistance. In contrast, pulmonary vascular resistance decreases due to spontaneous respiration opening the pulmonary vascular bed. The ductus arteriosus typically closes within 12 to 72 h after birth. An increase in the pulmonary blood flow and resulting increased left atrial filling shifts the flap of the foramen ovale reducing the atrial shunt size. Although complete closure of the foramen ovale may take a long time, the shunt through this small atrial opening is hemodynamically not significant. With the closure of the ductus arteriosus and foramen ovale, these changes result in the conversion of the parallel fetal circulation into the “in series” postnatal circulation [12] (Figure 1B)

### 1.2. Anticipated Acuity of Postnatal Symptoms in CHDs

Several factors are at play when predicting the risk of postnatal hemodynamic instability associated with CHD lesions. In CHDs, where abnormal anatomy results in a dependency on patency of the ductus arteriosus to provide/supplement either pulmonary or systemic blood flow, hemodynamic consequences of inadequate pulmonary or systemic perfusion can manifest within a few hours of life as the ductus arteriosus closure ensues. Acute hemodynamic instability can also occur if the heart cannot tolerate sudden increases in systemic vascular resistance resulting from cord clamping. If the pulmonary venous return is obstructed, such as in obstructed total anomalous pulmonary venous return (TAPVR) or hypoplastic left heart syndrome (HLHS) with intact/restrictive atrial septum, pulmonary blood flow and pulmonary venous return to the left atrium can also be acutely impaired. Non-cardiac abnormalities affecting the respiratory system may also play a role in hemodynamic instability, such as extreme lung hypoplasia, which can further compromise cardiopulmonary function in the immediate neonatal period.

## 2. Models for Risk-Stratification of Perinatal Care in Newborns with CHDs

Fetal echocardiography helps with the prenatal diagnosis of CHDs and serial evaluation during pregnancy, allowing prediction of the risk of hemodynamic instability in the immediate newborn period. Several authors have proposed methods for classifying prenatally diagnosed CHDs to stratify perinatal care requirements based on their fetal echocardiographic features [7,8,9,10,11]. Fetal cardiology guidelines published by the American Heart Association outline a risk-stratification scheme for perinatal and delivery room cardiac care using fetal echocardiographic criteria [13] as described below and summarized in Table 1.

### 2.1. CHDs without Predicted Risk of Hemodynamic Instability at Birth

This group includes “simple” CHD lesions such as mild tricuspid or pulmonary valve abnormalities, isolated left to right shunt lesions, and benign arrhythmias such as premature atrial complexes. Because these conditions are not expected to present any hemodynamic instability after birth, newborns with these conditions do not require specialized care after delivery. In these cases, the delivery planning can be based on the level of maternal care needs, and delivery can occur at a local hospital [14]. Management of CHDs in these newborns should include a transthoracic echocardiogram to confirm the diagnosis. In addition, if resources are available, cardiology consultation should be considered to counsel the parents regarding the CHD diagnosis and discuss future management plans. Based on the severity of the postnatal cardiac findings and pediatric cardiology resources available at the delivering hospital, this postnatal cardiology consultation can be obtained as an inpatient consult, telemedicine consult, or outpatient cardiology clinic visit within a few weeks of life. In some cases, the timing of the postnatal evaluation should be determined in light of common associated findings, e.g., coarctation in an atrioventricular canal defect, which may make pre-discharge cardiac evaluation preferable.

### 2.2. CHDs with Minimal or Low Risk of Hemodynamic Instability

Infants with CHD lesions reliant on patency of ductus arteriosus for either systemic or pulmonary circulation typically have lower pulse oximetry than normal infants. However, they are otherwise expected to be hemodynamically stable soon after birth. Suppose the ductus arteriosus closes between 12 to 72 h of life [15]. In that case, they may develop poor pulmonary circulation, resulting in profound cyanosis or poor systemic perfusion with hypotension and acidosis based on the CHD type. Although these lesions were considered critical CHDs [16] in the prior era, prenatal detection can prevent this hemodynamic instability by an opportunity to initiate PGE1 infusion soon after birth and, therefore, these ductal-dependent CHD lesions can be “re-categorized” as CHD with minimal/low risk of postnatal hemodynamic instability. These deliveries should have a neonatologist and necessary resources in the delivery room to start infusion of PGE through an umbilical venous line or a reliable peripheral intravenous line. After initial stabilization, they need transportation to a cardiac center for evaluation and potential cardiac surgical or catheter-based interventions. Additionally, they may require respiratory support, arrhythmia management, and other medical therapy. Hospital selection should consider local resources, proximity to a cardiac center, and parental preferences to minimize separation between mother and infant.

### 2.3. CHDs with a Moderate or High Risk of Hemodynamic Instability

These CHD lesions are expected to have moderate or severe hemodynamic instability soon after birth. Within the first few minutes or hours of life, these newborns require immediate neonatal stabilization, including lifesaving procedures and urgent cardiac intervention. The care team needs specialized knowledge of cardiac physiology in these complex CHDs for appropriate management. These specialized interventions may include cardiac surgical interventions, catheterization procedures, arrhythmia management, or initiation of extracorporeal membrane oxygenation. Therefore, these complex CHD deliveries should be planned at tertiary hospitals with the availability of appropriate resources based on the anticipated needs of the infant. The mode and timing of delivery are determined by the multidisciplinary care team, including a maternal–fetal medicine specialist, neonatologist, fetal cardiologist, pediatric cardiac intervention specialist, other pediatric specialists, and surgeons. Planned induction of labor or cesarean section prior to 40 weeks gestation is often proposed so that the recommended postnatal care plan is coordinated and instituted in a timely fashion [8,17,18]. The individual lesion-specific delivery planning section describes further details and special considerations for the delivery planning, and this information is summarized in Table 2.

## 3. Delivery Planning Based on CHD Lesion-Specific Risk-Stratification (Table 2)

### 3.1. Coarctation of the Aorta

Prediction of the presence or severity of aortic arch coarctation is challenging prenatally due to the presence of the ductus arteriosus, with its true severity often becoming evident only after the ductal constriction. Several studies have evaluated the utility of fetal echocardiographic findings in the prediction of postnatal coarctation, such as ventricular size discrepancy [19], aortic isthmus hypoplasia using gestational age-specific Z scores [20,21], increased distance between left common carotid to left subclavian branches, aortic arch curvature [22], and retrograde flow in the aortic isthmus [23]. However, none of these individual markers had high sensitivity and specificity for accurate coarctation prediction, and multi-parametric models integrating different fetal echocardiographic variables were proposed after a metanalysis study [24]. A more recent metanalysis study [21] again showed that although several prenatal ultrasound parameters can be associated with coarctation, their diagnostic accuracy is only moderate, even when parameters were used in combination. It has been reported that risk-based delivery and perinatal care planning in suspected coarctation can reduce unnecessary medical interventions [25]. Low-risk cases can be delivered locally utilizing standard postnatal care protocols with a planned postnatal echocardiogram before discharge and a cardiology consult or follow-up based on echo findings. High-risk cases are usually recommended for PGE1 initiation at delivery and transfer to a cardiac center. In cases where the diagnosis of coarctation cannot be ruled out on the initial fetal echo due to poor image resolution but intracardiac anatomy was reassuring, obtaining a neonatal transthoracic echocardiogram to evaluate the aortic arch after birth without doing a follow-up fetal echocardiographic evaluation is the most cost-effective approach [26]. This severity-based tiered postnatal management approach was cost-effective and safe in achieving timely treatment of coarctation in those who needed it while reducing resource utilization in those who did not develop postnatal coarctation [7].

### 3.2. Tetralogy of Fallot

Tetralogy of Fallot (ToF) is the most common cyanotic CHD. Most lesions are detected prenatally, with a prenatal detection rate of 90% of patients in experienced centers [27]. For ToF patients where the pulmonary valve is present, the degree of right ventricular outflow tract obstruction and dependency on ductus arteriosus for adequate pulmonary blood flow determines the severity of cyanosis after birth. Fetal echocardiographic features that can help predict ductal-dependent pulmonary blood flow include flow reversal in the ductus arteriosus and a small size of the pulmonary valve. Reversed flow at the ductus arteriosus strongly predicts postnatal ductal dependency [7,28,29]. Additionally, the severity of pulmonary valve hypoplasia correlates with a higher risk of ductal-dependent pulmonary blood flow. For example, a pulmonary valve Z score of less thannegative 3 had a 100% sensitivity though only 34% specificity for predicting a ductal-dependent circulation. In comparison, the pulmonary valve Z score of less than −5 had a sensitivity of 78% and increased the specificity to 87%. A pulmonary valve-to-aortic valve ratio of less than 0.6 and a pulmonary valve-to-aortic valve Z score difference of ≥5 were at higher risk for ductal dependency [29].

Given these findings, fetuses with a prenatal diagnosis of ToF suggestive of ductal-dependent pulmonary blood flow should be considered to be delivered close to a cardiac center and have resources available for starting PGE infusion soon after birth. These newborns can then be safely transferred to the cardiac unit for further management and neonatal surgical or catheter-based intervention. For those without suspicion of ductal-dependent pulmonary blood circulation, the plan may include postnatal confirmation of the diagnosis by transthoracic echocardiography, cardiology consultation as needed, outpatient follow-up, and elective repair of the ToF at a few months of age. ToF with an absent pulmonary valve is discussed separately.

### 3.3. Hypoplastic Left Heart Syndrome

Survival in hypoplastic left heart syndrome (HLHS) requires ductus arteriosus patency to maintain systemic blood flow and sufficient interatrial communication to allow pulmonary venous return from the left to the right atrium. However, 6–20% of HLHS newborns exhibit restrictive or intact atrial septum (RAS) [30,31]. RAS in the setting of HLHS results in impairment of pulmonary venous egress, leading to pulmonary edema, severe hypoxemia, and poor cardiac output within minutes after birth. Urgent cardiac intervention to open the atrial septum is necessary for survival, allowing pulmonary venous blood flow to egress out of the left atrium. Although the mortality in HLHS with RAS is higher than in HLHS with unobstructed atrial septal communication, survival can be potentially improved if the atrial restriction is detected prenatally to allow for planning an urgent cardiac intervention to open the atrial septal communication soon after birth [30].

Fetal echocardiographic findings such as an abnormal pulmonary venous Doppler can predict RAS in HLHS [32,33,34,35]. The strongest fetal echocardiographic predictor for postnatal RAS and the need for emergent atrial septoplasty is a pulmonary venous Doppler forward/reverse velocity time interval ratio. A VTI ratio of <5 predicts RAS and the need for atrial septal intervention with a 100% sensitivity [36]. Furthermore, a ratio of <3 can predict an RAS and need for emergent atrial septoplasty with 94% specificity, eliminating any false positive diagnosis [37]. A more recent study evaluating 68 fetuses with critical left heart obstruction and restrictive or intact atrial septum showed that the strongest discriminators for severe neonatal illness were a pulmonary vein VTI ratio of ≤2.7 and a larger pulmonary vein diameter [38]. As the atrial septal restriction can progress in the third trimester, serial fetal echocardiographic assessment is recommended, including late gestation (>35 weeks gestation) assessment specifically focusing on the atrial septum and pulmonary venous flow pattern [10]. In addition to standard fetal echo, the use of acute maternal hyperoxia testing (MHO) may be a useful adjunct in the assessment of pulmonary vasculopathy in fetuses with HLHS [39]. The utility of MHO is discussed separately under newer techniques in fetal CHD assessment.

Additionally, a fetal MRI can also be considered if such a resource is available, as the presence of pulmonary lymphangiectasia, described as the “nutmeg lung” pattern, has been associated with a more severe presentation and significant mortality risk [40,41].

Even if the atrial septum appears unobstructed based on those earlier mentioned fetal echocardiographic criteria, all fetuses with prenatally detected HLHS require specialized postnatal care. Their delivery should be planned in a medical center where neonatal care resources are available, such as the presence of a neonatologist in the delivery room, availability of PGE1 infusion, and resources for mechanical ventilation if needed. Supplemental oxygen can reduce pulmonary resistance and compromise systemic perfusion in HLHS. Therefore, supplemental oxygen should be used cautiously. After initial stabilization, if the delivering hospital lacks congenital cardiac surgical capabilities, the newborns must be promptly transported to the appropriate center. Longer transport time is associated with higher pre-transport and pre-surgical mortality [42]. The timing and mode of delivery in these cases should be based on the obstetrical history and indications aiming for delivery at or near term, as overall outcomes in HLHS are better for deliveries after 39 weeks of gestation than those born before 37 weeks of gestation [43].

Fetuses with HLHS and RAS need much more specialized multidisciplinary delivery planning and care coordination between the obstetrical and pediatric specialists needed at the time of birth to allow immediate cardiac interventions. These specialists include neonatologists, pediatric cardiologists, pediatric cardiac interventionalists, cardiac anesthesiologists, and cardiothoracic surgeons. In some instances, cesarean section may facilitate coordination of this multispecialty care. Parents should be presented with the potential benefits of a timed delivery with a cesarean section, allowing for appropriate preparedness of the cardiac team vs. the potential risks associated with this mode of delivery to arrive at shared decision-making (Table 1).

### 3.4. Total Anomalous Pulmonary Venous Return (TAPVR)

TAPVR is challenging to diagnose prenatally with a very low prenatal detection rate of <2% in series before 2004 [44] and only 12–13% in more recent studies [45,46,47,48]. When TAPVR is known prenatally, delivery planning should be based on the risk of postnatal obstruction of the TAPVR based on fetal echo findings. Although prenatal hemodynamics can underestimate the degree of vertical vein obstruction, certain fetal echocardiographic features can help stratify the risk of TAPVR obstruction after birth, such as fetal vertical vein Doppler peak velocity of >0.74 m/s mmHg [47], reduced pulsatility of flow in the vertical vein [49], or anatomic factors such as infra-diaphragmatic type of TAPVR. It is important to consider that obstruction of TAPVR may be underappreciated on a fetal echocardiogram due to the low amount of pulmonary blood flow in utero, causing false reassurance. The use of acute MHO testing may help uncover obstruction of TAPVR drainage once the pulmonary blood flow increases in response to oxygen. It is reasonable to consider the delivery of such a fetus at a cardiac center or nearby in preparation to be able to do urgent cardiac interventions to address the obstructed TAPVR. Traditionally, obstruction of TAPVR was addressed by urgent surgical interventions. However, few centers have tried the cardiac catheterization-based approach of balloon or stent placement within the vertical vein draining the TAPVR to temporarily stabilize the newborn before addressing the TAPVR by surgical repair later in time [50]. Unless there are obstetrical or additional fetal concerns, delivery prior to 39 weeks should be avoided. In some instances, a cesarean section may facilitate coordination of multispecialty care.

### 3.5. D-Transposition of the Great Arteries with Restrictive Atrial Septum

D-TGA results in parallel systemic and pulmonary circulation rather than normal circulation in series. Therefore, tissue oxygenation relies on wide open atrial-level communication to adequately mix the oxygenated and deoxygenated blood. In case there is a restriction of this atrial-level shunt, significant hypoxemia can result after birth. In that case, these newborns require the cardiac catheterization procedure to open up the atrial septum by balloon atrial septostomy (BAS), and failure to do so can result in severe hemodynamic decompensation and poor outcomes, including death [51,52,53]. Although prenatal diagnosis of D-TGA is increasing, data regarding accurate fetal echocardiographic predictors of postnatal atrial septal restriction in D-TGA are limited. Fetal echocardiographic findings that can suggest restriction of the foramen ovale include the small size of the atrial septal opening in comparison to the total atrial septal length, hypermobile atrial septum that moves into the right and left atrium, ductal flow restriction, and higher Doppler flow velocity in pulmonary veins. Although these fetal echocardiographic features can predict a higher risk of postnatal atrial septal restriction with an overall good specificity, the sensitivity is low. Therefore, the absence of these features cannot effectively rule out the possibility of needing BAS after birth [7,51,54]. Given this low sensitivity and inability to effectively rule out possible need for BAS, all newborns with D-TGA are recommended to have a high-risk delivery plan. These fetuses should be delivered at a cardiac center to allow for rapid postnatal management. Induction of labor after 39 weeks gestation may allow for a more planned delivery with resource preparation for the urgent BAS. Although it is rare if the foramen ovale is closed and the ductus arteriosus is severely restrictive, predicting severe postnatal hypoxemia, even a cesarean section may be considered for accurately timed delivery with all the resources for BAS on standby. A recent study additionally supports this argument for a timed cesarean delivery showed that as compared to birth during regular work hours, out-of-hours deliveries were associated with longer time to BAS and higher neurological morbidity, especially in those with a restrictive septum, making an argument for timed delivery via cesarean, especially if there are concerns for atrial septal restriction [55]. In addition to the planning for BAS, additional resources are required for neonatal resuscitation and stabilization, such as the availability of PGE infusion and mechanical ventilation using supplemental oxygen to reduce hypoxia as much as possible. Newborns with D-TGA are also at a higher risk for extreme pulmonary hypertension, resulting in reversed cyanosis. Treating pulmonary hypertension with 100% oxygen and inhaled nitric oxide may benefit these cases.

### 3.6. Ebstein Anomaly and Tricuspid Valve Dysplasia

Ebstein anomaly or dysplasia of the tricuspid valve is a rare CHD. The Ebstein anomaly can result in atrialization of the right ventricle, reducing effective right ventricular size, and severe tricuspid regurgitation, resulting in right atrial enlargement and massive cardiomegaly. These changes can lead to fetal heart failure, hydrops, and fetal arrhythmia [56]. In addition, massive cardiomegaly occupies much of the intrathoracic space and can lead to lung hypoplasia. Due to these multiple possible complications, these tricuspid valve anomalies are associated with severe hemodynamic instability after birth and very high perinatal mortality rates. A prior multi-center retrospective study showed a perinatal death rate of up to 45% in prenatally diagnosed Ebstein anomaly of tricuspid dysplasia cohort [57]. Fetal echocardiographic risk factors for perinatal mortality in this study included findings of pulmonary valve regurgitation, larger tricuspid valve annular Z score, pericardial effusion, and ventricular dysfunction. A “circular shunt “resulting from a combination of retrograde flow in the ductus arteriosus, pulmonary regurgitation, and severe tricuspid regurgitation is a particularly severe presentation associated with a high risk for hemodynamic instability [58,59]. In addition, prematurity with delivery at <32 weeks gestation was also a risk factor for perinatal mortality. As no prospective studies evaluate outcomes with specific delivery management strategies in these lesions, their perinatal management often varies between institutions [57]. However, considering that Ebstein anomaly is associated with lung hypoplasia, delivery should be delayed until the near term to allow for optimal lung maturation unless there is an indication for earlier delivery due to evidence for fetal compromise such as fetal hydrops, severe cardiac dysfunction, or uncontrolled arrhythmia [13]. On the other hand, some centers have advocated for elective early delivery, particularly in the case of circular shunts, to allow for early surgical intervention with ductal ligation [60], given the risk for IUFD in these fetuses.

### 3.7. Tetralogy of Fallot with Absent Pulmonary Valve (ToF/APV)

The pulmonary valve can be functionally absent in up to 6% of fetuses diagnosed with tetralogy of Fallot [61]. The to and fro flow across the right ventricular outflow tract due to the absence of a pulmonary valve may result in severe main and branch pulmonary artery dilation. A dilated pulmonary arterial tree can cause extrinsic airway compression and is often associated with airway abnormalities such as congenital lobar emphysema, tracheobronchial obstruction, and pulmonary hypoplasia. ToF/APV can develop right-sided cardiac failure, hydrops, and even in utero fetal demise. Airway compression may lead to severe hypoxia after birth, resulting in perinatal mortality and morbidity, with reported perinatal death rates up to 42% [61,62,63]. This is typically not a ductal-dependent lesion, and even in utero, it often lacks a ductus arteriosus [64]. Fetal echocardiographic predictors have not been consistently reliable in accurately predicting postnatal outcomes. However, mediastinal shift of the cardiac mass and ventricular dysfunction were associated with higher mortality in a recent multi-center study [62,65]. Recently, the utility of fetal MRI was evaluated in predicting postnatal outcomes. Fetal MRI findings of fluid trapping in the lungs due to severe airway obstruction in fetuses with ToF/APV correlated with poor neonatal outcomes [66,67]. They may indicate a need for more proactive management of severe airway insufficiency in the immediate postnatal period.

Close fetal monitoring is indicated in ToF/APV due to the risk of fetal distress and hydrops. Early delivery may be recommended if fetal cardiac failure or hydrops fetalis is noted. Delivery should be planned at a cardiac center or near a cardiac center in order to plan a rapid newborn transport to the cardiac center. Preterm delivery should be avoided to allow for optimal lung maturation unless fetal distress indicates early delivery.

If respiratory distress due to airway compression from dilated pulmonary arteries and abnormal pulmonary vasculature occurs in newborns with ToF/APV, placing the infant in a prone position can help relieve pressure on the bronchus. Additionally, mechanical ventilation may be necessary. Inhaled nitric oxide and 100% oxygen can reduce pulmonary vascular resistance and enhance forward pulmonary flow. Newborns with extreme distress may require extracorporeal membrane oxygenation support. Since this is not a ductal-dependent lesion, PGE is not indicated. Additionally, genetic testing is recommended to evaluate for possible 22q11 deletion for these patients, either prenatally or after birth to guide future management of non-cardiac-associated findings seen in 22q11 deletion syndrome.

### 3.8. Uncontrolled Tachyarrhythmia

Fetal tachyarrhythmias such as reentry tachycardia or atrial flutter have a risk of fetal distress, hydrops, and perinatal mortality. Transplacental treatment of fetal tachycardia by administering anti-arrhythmia medications through the mother has a high success rate of arrhythmia control, even in the presence of hydrops [68]. However, refractory cases with fetal hydrops may need early delivery. The cesarean section is indicated in these cases as fetal monitoring to detect fetal distress during vaginal delivery is not possible due to ongoing fetal tachyarrhythmia. Ongoing arrhythmia at birth can cause significant hemodynamic compromise, especially if there were in utero hydrops leading to a decision to deliver. Therefore, delivery should be planned at or near a cardiac center. Neonatologists should collaborate with pediatric electrophysiologists to prepare anti-arrhythmia medications and plans to stabilize the newborn in the delivery room, followed by prompt transfer to the cardiac center for further neonatal cardiac management.

### 3.9. Congenital Complete Heart Block

Congenital complete heart block (CHB) causes significant fetal bradycardia. The most common etiology for CHB is immune-mediated damage due to exposure to maternal SSA and SSB antibodies on the developing fetal conduction system, resulting in complete heart block. Another etiology is the abnormal development of the conduction tissue associated with certain congenital heart defects such as left atrial isomerism and heterotaxy or atrioventricular septal defect [69,70,71,72]. Regardless of the etiology of CHB, it results in significant fetal bradycardia, reduced combined cardiac output, increased risk for fetal hydrops, and an overall high perinatal mortality rate [69,70,71,72]. Findings of endocardial fibroelastosis, placental infarction, fetal heart rate (ventricular rate) of less than 50 bpm [73], or development of hydrops fetalis are considered risk factors associated with perinatal mortality in CHB cases.

Close fetal monitoring using cardiovascular profile scores and intermittent evaluation with biophysical profile scores is recommended in cases of CHB. If there is evidence of fetal distress and deteriorating cardiac performance, early delivery may be necessary even in cases where there is prematurity [70]. Neonatal bradycardia can result in poor cardiac output due to bradycardia requiring emergent pacemaker insertion during the neonatal period. Fetuses with a fetal heart rate < 50 bpm have a very high risk of needing neonatal pacemaker placement [74]. Therefore, delivery of a fetus with CHB and low FHR is recommended at the cardiac center or in proximity to resources such as the presence of neonatologists and cardiologists in the delivery room, resources for urgent therapy such as chronotropic agents and inotropic agents, as well as the ability to initiate temporary pacing.

## 4. Other Considerations in Delivery Planning

### 4.1. Mode of Delivery in CHD Fetuses

While a select group of high-risk CHD cases may necessitate delivery by a cesarean section to ensure fetal well-being or cardiac team availability, vaginal delivery is safe for most of the other CHD lesions. Studies have shown that rates of cesarean sections and risk of hemodynamic complications in the delivery room for complex CHD fetuses are comparable to healthy fetuses with the help of multidisciplinary care coordination and the use of standardized delivery room management protocols [9]. Although the risk of neonatal acidosis in CHD infants is similar to non-CHD infants for shorter duration of vaginal deliveries, prolonged vaginal delivery in CHD fetuses showed declining median umbilical cord blood Ph as labor progressed [75]. Therefore, prolonged duration of labor during vaginal delivery can imply a risk of hemodynamic instability in newborns after birth as a potentially modifiable factor to improve CHD outcomes.

### 4.2. Strategies to Protect the Brain during Transitional Circulation

Newborns with CHDs are particularly susceptible to brain vulnerability during the transitional period during and immediately after delivery, either due to hypoxemia or poor cardiac output. Several management strategies are proposed to support cerebral flow and oxygenation and potentially influence neurodevelopmental outcomes in this population [76]. It is recommended to minimize premature or even early-term delivery, as the immature brain may be more susceptible to hypoxemia. Studies comparing cesarean versus vaginal delivery for CHD fetuses have shown no significant differences in perinatal hospital length of stay, prevalence of perioperative morbidities, or death prior to discharge [16]. Minimizing hypoxia in the delivery room and immediate neonatal period is crucial for reducing the risk of perioperative brain injury. Adequate resource planning to ensure appropriate neonatal resuscitation can help avoid significant postnatal hypoxia [17].

### 4.3. Utility of Acute Maternal Hyperoxia Testing in Improving Risk-Stratification

Acute maternal hyperoxia (MHO) testing can be performed as an adjunct to standard fetal echocardiography by evaluating select fetal echocardiographic variables after maternal administration of inhaled oxygen for 10 to 15 min through a non-rebreather face mask. This short-term maternal exposure to inhaled oxygen and resulting changes in the fetal pulmonary Doppler blood flow pattern can help characterize fetal pulmonary vasoreactivity to oxygen and help predict postnatal physiology in certain CHDs with a high risk of hemodynamic instability [77,78,79,80]. When performed in late gestation (usually after 34 weeks gestation), MHO increases circulating oxygen content in the fetal blood, eliciting a physiologic response in the fetus characterized by a temporary increase in pulmonary blood flow [81]. Normal fetal pulmonary vasoreactivity in response to MH is characterized by a ≥10% decrease in the Doppler pulsatility indices [(peak systolic velocity − end-diastolic velocity)/mean velocity] in the branch pulmonary arteries [78,81]. Although there is a good amount of data related to using MHO in HLHS in the prediction of restrictive atrial septum, only limited data exist for using MHO in other CHD lesions. The fetal pulmonary vasoreactivity and anatomic and physiologic changes noted in the fetus with MH were predictive of postnatal findings and the need for urgent cardiac interventions in a study that included 12 fetuses with Ebstein anomaly, total anomalous pulmonary venous connection, HLHS with restrictive or intact atrial septum, and D-TGA [78]. A systemic review of the utility of MHO evaluating nine studies did not show any significant maternal adverse reactions, and acute MH was successfully used to risk-stratify HLHS fetuses in three of these studies [82]. In a recent study of HLHS fetuses, assessing changes in pulmonary venous flow parameters with MHO was found to discriminate fetuses with “intermediate” pulmonary vein flow profiles, and pulmonary venous flow assessment was found to be more reproducible than pulmonary artery pulsatility index [83]. Further studies are needed to define the utility of MHO in accurately predicting risk for hemodynamic instability in CHDs soon after birth.

### 4.4. Cardiovascular Profile Score

Cardiovascular profile score (CVP) is a useful tool for fetal assessment. A low CVP or progressive decline of CVP score can predict the risk of in utero demise. In CHD fetuses with a risk for cardiac dysfunction or poor cardiac output and heart failure, serial monitoring with CVP can help determine the need for early delivery if the risk of in utero demise is high [84]. The CVP score is derived using five fetal echocardiographic markers: signs of hydrops, cardiac size, ventricular systolic function, and umbilical arterial and venous Doppler flow pattern. These parameters are rated from 0, 1, and 2 based on normal findings, mild abnormality, and significant abnormality, respectively. Therefore, the final score ranges from 10 (normal CVP) to 0. CHD fetuses with CVP ≤ 7 on the last fetal echo prior to delivery had a higher risk of perinatal mortality and worse postnatal outcomes compared to those with CVP > 7 [84]. A systemic review evaluating CVP in predicting fetal outcomes confirmed that a CVP ≤ 7 predicted statistically significant adverse fetal outcomes, and the significance further increased at CVP ≤ 6 [85]. Another recent study by Miyoshi et al. indicated that CHD fetuses with CVP ≤ 7 were more likely to require urgent cesarean delivery due to non-reassuring fetal status as compared to CHD fetuses with CVP ≥ 8 [86].

### 4.5. Multidisciplinary Approach for Delivery Planning

Multiple stakeholders are involved in the planning of delivery and neonatal resuscitation of a CHD fetus, such as fetal cardiologists, obstetricians, maternal–fetal medicine specialists, neonatologists, and cardiac intensivists. Additional team members may be included based on the anticipated need for cardiac interventions during the neonatal period, such as cardiac catheterization or cardiovascular surgical teams. Moreover, this team may also include palliative care professionals, social workers, and others who provide comprehensive and supportive family-centered care. Planning for delivery in these high- or very high-risk CHD infants should consider factors such as the predicted risk of hemodynamic instability at birth, availability of medical resources in the region, proximity to the nearest cardiac care center, the type and availability of the transportation to the cardiac center, and anticipated or known obstetric or maternal complications [87].

## 5. Conclusions

Prenatal detection of CHDs is increasing, owing to the advances in fetal cardiac imaging techniques and improved obstetrical screening protocols identifying at-risk fetuses that are referred for further evaluation by fetal echocardiography. Fetal echocardiographic features help predict the severity of postnatal hemodynamic instability after birth. Therefore, serial fetal echocardiographic assessment can guide the planning for appropriate neonatal stabilization and cardiac interventions for each CHD fetus. A multidisciplinary approach is required to create and execute CHD lesion-specific, individualized delivery and perinatal management plans for CHDs with a high risk of hemodynamic instability and the potential need for urgent cardiac interventions. Newer techniques, such as acute maternal hyperoxia testing, may assist in more accurately predicting anticipated postnatal compromise in certain lesions where such prediction remains challenging.

**Table 1:** Suggested risk-stratified classification of postnatal care of newborns with CHDs.

Predicted Risk of Hemodynamic Instability at Birth	Example of CHD	Suggested Classification System	Suggested Delivery Plan *	DR Recommendations	Anticipated Neonatal Management in the First Few Days of Life
None/Minimal	Simple shunt lesions, such as isolated VSD and balanced AVSD, mild valve disease, benign arrhythmias.	LOC 1	Mode and time of delivery: Based on the level of maternal care.	There is no specialized care in the DR. Notify the primary care provider of the fetal echo findings and cardiology recommendations for postnatal management.	May room in with mother or stay in the newborn nursery, obtain echo prior to discharge, consider cardiology consult **, or arrange outpatient cardiology evaluation.
Low	Ductal-dependent lesions: HLHS, HRHS variants, tricuspid atresia, interrupted aortic arch, critical aortic stenosis, critical pulmonary stenosis, PA/IVS, ToF with severe RVOTO, non-sustained or controlled tachyarrhythmias or bradyarrhythmias with adequate ventricular rate.	LOC 2	Mode and time of delivery are based on the level of maternal care. Planned delivery ≥ 39 wks can be considered to coordinate services needed to manage a CHD newborn.	A neonatologist should be present in the DR. Place UA and UV lines, stabilize, start PGE if indicated based on CHD lesion, and transfer to a cardiac center for further management.	Admit to NICU or CICU. Arrange for neonatal cardiac interventions (catheterization or surgery) as needed.
Moderate	HLHS at risk for RAS, D-TGA at risk for RAS, CHD or arrhythmia with decreased heart function, CHD or arrhythmia with hydrops.	LOC 3	Mode and time of delivery: Planned delivery ≥ 39 wks should be arranged to coordinate service.	The neonatologist and cardiologist in the DR and cardiac services were alerted. Plan for intervention or urgent transport if indicated.	Transport to cardiac center. Admit to NICU or CICU. Arrange for catheterization or surgery.
High	HLHS with severe RAS or IAS, TGA with severe RAS or IAS, abnormal DA shunt, obstructed TAPVR tachy- or brady- arrhythmia with hydrops, severe Ebstein anomaly with hydrops, ToF/APV.	LOC 4	Mode and time of delivery: Planned delivery ≥ 39 wks (or earlier if fetal cardiac dysfunction or hydrops are suspected and GA appropriate) at the cardiac center should be arranged.	The neonatologist, cardiologist, and surgery team in the DR. Plan for intervention (catheterization, surgery, or ECMO).	

AVSD, atrioventricular septal defect; CHD, congenital heart disease; DA, ductus arteriosus; DR, delivery room; D-TGA, transposition of the great arteries; ECMO, extracorporeal membrane oxygenation; GA, gestational age; HLHS, hypoplastic left heart syndrome; IAS, intact atrial septum; LOC, level of care; PA/IVS, pulmonary atresia with intact ventricular septum; PGE, prostaglandin E; RAS, restrictive atrial septum; TAPVR, total pulmonary venous return; ToF, tetralogy of Fallot; ToF/APV, tetralogy of Fallot with absent pulmonary valve; VSD, ventricular septal defect. * The delivery plan may vary according to LMC, the pediatric resources of the delivery hospital, the distance to the cardiac care center, and the type and availability of transportation to the cardiac center. The delivery plan should ensure the appropriate DR recommendations. ** In centers without a cardiologist, this consult can be done via telemedicine or an early outpatient cardiology visit should be arranged.

**Table 2:** CHD lesion-specific prenatal predictors of postnatal hemodynamic instability and suggested delivery room care recommendations.

CHD Diagnosis	Fetal Echocardiogram Findings	Delivery Room (DR) Recommendations
**ASD, VSD, or AVSD (shunt lesions);** **mild valve abnormalities**	Isolated ASD or VSD with normal FO and DA flow, normal or minimal flow disturbances at valves, and normal heart function (LOC 1).	Routine care, hospital, or telemedicine consult. Outpatient cardiology follow-up.
**Coarctation, critical** **(ductal-dependent lesion)**	Ductal-dependent coarctation [21] (LOC 2): Left/right heart size discrepancy with MV/TV and AoV/PV ratios < 0.6 [52]. Distal arch in 3rd trimester < 3 mm [52]. AoI/DA in 3VV < 0.75 [53]. Abnormal Doppler flow in isthmus [13,54] retrograde flow in the aortic isthmus [23]. Posterior shelf [56]. Aortic isthmus hypoplasia using gestational age-specific Z scores [20]. Carotid subclavian index (increased distance between left common carotid to left subclavian branches). Aortic arch curvature [22].	Initiation of prostaglandin infusion through peripheral IV or umbilical line. Intubation with mechanical ventilation only if clinically indicated. Transfer to a cardiac center.
**Pulmonary atresia, HLHS, other single ventricles, or cyanotic ToF** **(ductal-dependent lesions)**	Ductal-dependent pulmonary circulation (LOC 2): Aorta to pulmonary flow in the DA [37,38]. Reversed orientation of the DA (inferior angle < 90°) [39]. Pulmonary valve Z score value less than 3 after 16 weeks [40]. Ductal-dependent systemic circulation (LOC 2): Left to right atrial flow across the foramen ovale and distal aortic arch [37,41]	Initiation of prostaglandin infusion through peripheral IV or umbilical line. Intubation with mechanical ventilation only if clinically indicated. Transfer to a cardiac center.
**HLHS and variants with severely restrictive or intact atrial septum**	Pulmonary vein Doppler [42,43]: Moderate obstruction: PV f/r < 5 and >3 (LOC 3). Severe obstruction: PV f/r < 3; (LOC 4).	Initiation of prostaglandin infusion through peripheral IV or umbilical line Intubation with mechanical ventilation OR or Cath lab on standby. Plan for immediate intervention to decompress the left atrium. ECMO is available.
**Obstructed TAPVR**	Pulmonary vein Doppler [40]: Monophasic non-pulsatile pulmonary venous flow (LOC 4). Reduced pulsatility in vertical venous flow [49]. Infra-diaphragmatic TAPVR (LOC 3 or 4).	Intubation with mechanical ventilation. Peripheral IV and/or umbilical line OR team on standby. Initiation of prostaglandin infusion (may relax the ductus venosus smooth muscle for infra-diaphragmatic TAPVR). Plan for immediate surgical intervention.
D-TGA and variants with restrictive atrial septum *	Foramen ovale findings [45,46]: Hypermobile septum (LOC 3). Angle of septum primum < 30° (LOC 3). Lack of swinging motion of septum or “tethered” septum (LOC 3). Bowing of atrial septum >50% (LOC 3 or 4). Small FO (FO/total septal length < 0.30) or intact (LOC 4). Abnormal ductus arteriosus ([47,48]): With small FO < 3 mm (LOC 4). Small ductus arteriosus (<3 mm) with moderate/severe restriction (LOC 4). Reversed, bidirectional, or accelerated flow (LOC 3). Pulmonary vein Doppler, proximal to the left atrium [49]: Max velocity “s” wave > 41 cm/s (LOC 3 or 4).	Initiation of prostaglandin infusion through peripheral IV or umbilical line. Intubation with mechanical ventilation. Cath lab on standby. Plan for immediate balloon atrial septostomy. If ductal flow is abnormal, consider pulmonary hypertension therapy, including intubation, 100% oxygen, and inhaled nitric oxide.
**ToF/APV**	With associated cardiac dysfunction (LOC 3). With associated hydrops fetalis (LOC 4). Lung findings suggestive of compression or fluid trapping (LOC 4).	Specialized cardiac care team in the DR. Specialized ventilation (prone). Peripheral IV or umbilical access. Intubation with mechanical ventilation if needed. Consider 100% oxygen and inhaled nitric oxide to decrease pulmonary resistance. Consider ECMO.
**Severe Ebstein’s anomaly**	With associated cardiac dysfunction (LOC 3). With associated hydrops fetalis (LOC 4).	Specialized cardiac care team in the DR. Specialized ventilation (prone). Peripheral IV or umbilical access. Intubation with mechanical ventilation if needed. Consider 100% oxygen and inhaled nitric oxide to decrease pulmonary resistance. Consider ECMO cardioversion or medical therapy in DR as indicated for arrhythmia.
**Unstable tachyarrhythmias**	With associated heart failure (LOC 3). With associated hydrops fetalis (LOC 4).	Consider early delivery if gestational age-appropriate. Cardioversion and/or medical therapy in DR.
**Complete heart block with low ventricular rate and/or cardiac dysfunction**	With associated heart failure (LOC 3). With associated hydrops fetalis (LOC 4).	Consider early delivery if gestational age-appropriate. Consider chronotropic agents vs. temporary pacing in DR.

ASD, atrial septal defect; AoI, aortic isthmus; AoV, aortic valve; APV, absent pulmonary valve; AVSD, atrioventricular septal defect; DA, ductus arteriosus; D-TGA, D-transposition of the great arteries; DR, delivery room; ECMO, extracorporeal membrane oxygenation; FO, foramen ovale; HLHS, hypoplastic left heart syndrome; IV, intravenous; LOC, level of care; MV, mitral valve; OR, operating room; PV f/r, pulmonary vein forward/reversed flow integral; PV, pulmonary valve; TAPVR, total abnormal pulmonary venous return; ToF, tetralogy of Fallot; TV, tricuspid valve; VSD, ventricular septal defect; 3VV, three-vessel view. * Fetal echocardiographic findings associated with higher risk for urgent BAS have high specificity but low sensitivity based on limited data.

## Data Availability

No data available for this review article.
